# Measures of diabetic retinopathy treatment coverage: protocol for a methodological review

**DOI:** 10.1136/bmjopen-2024-092081

**Published:** 2025-03-29

**Authors:** Nimisha Chabba, Pushkar Raj Silwal, Covadonga Bascaran, Rinki Murphy, Iris Gordon, Nyawira Mwangi, Subash Bhatta, Nayana Pant, Matthew J Burton, Stuart Keel, Jennifer Evans, Jacqueline Ramke

**Affiliations:** 1 School of Optometry and Vision Science, University of Auckland, Auckland, New Zealand; 2 International Centre for Eye Health, London School of Hygiene & Tropical Medicine, London, England, UK; 3 School of Medicine, The University of Auckland, Auckland, New Zealand; 4 Auckland Diabetes Centre, Greenlane Clinical Centre, Te Whatu Ora Health New Zealand Te Toka Tumai Auckland, Auckland, New Zealand; 5 Specialist Weight Management Service, Te Mana Ki Tua, Te Whatu Ora Health New Zealand Counties Manukau, Auckland, New Zealand; 6 Kenya Medical Training College, Nairobi, Kenya; 7 Pacific Eye Institute, Suva, Fiji; 8 Drishti Eye Care Hospital, Kathmandu, Nepal; 9 National Institute for Health Research Biomedical Research Centre for Ophthalmology, Moorfields Eye Hospital NHS Foundation Trust and UCL Institute of Ophthalmology, London, UK; 10 Department of Noncommunicable Diseases, World Health Organization, Geneva, Switzerland

**Keywords:** Diabetic retinopathy, DIABETES & ENDOCRINOLOGY, OPHTHALMOLOGY

## Abstract

**Abstract:**

**Introduction:**

Diabetic retinopathy is one of the leading causes of vision impairment globally. Alongside the systemic control of diabetes and timely detection of diabetic retinopathy, the prompt initiation and completion of treatment is essential to prevent vision loss. Routine monitoring of access to retinal screening services for the detection of diabetic retinopathy is common, while monitoring of coverage of subsequent treatment services is far less common. When diabetic retinopathy treatment coverage is assessed, there is great variability in how it is defined and reported. If a definition of treatment coverage could be standardised, the monitoring of the quality of diabetes eye care could more readily be compared between settings and over time. The aim of this review is to summarise how diabetic retinopathy treatment coverage has been measured in published studies and the extent to which these have been disaggregated by population groups.

**Methods and analysis:**

A search will be conducted on Medline and Embase without any language restrictions, for cohort and cross-sectional studies published from 1 January 2015 that report diabetic retinopathy treatment coverage for adults with diabetic retinopathy and/or macular oedema. We will include studies from any world region reporting diabetic retinopathy treatment coverage for one or more of: (1) laser photocoagulation; (2) intravitreal injections of antivascular endothelial growth factor agents; (3) intravitreal injections of corticosteroids; (4) vitrectomy. The PROGRESS framework (place of residence, race/ethnicity/culture/language, occupation, gender/sex, religion, education, socioeconomic status and social capital) will be used to assess disaggregation by population groups. Two investigators will independently screen studies and extract relevant data. Data will be synthesised descriptively to outline the full range of definitions of diabetic retinopathy treatment coverage in the literature and identify the common sources of data used.

**Ethics and dissemination:**

This review will only include published data; thus, no ethical approval will be sought. The findings of this review will be published in a peer-reviewed journal and presented at relevant conferences. The findings will also be considered in conjunction with an ongoing review on retinal screening for diabetic retinopathy to develop indicators for monitoring of services along the diabetes eye care pathway, which may include an indicator of effective service coverage.

**Registration:**

Open Science Framework registration 6/08/2024: https://osf.io/5b93m

**STRENGTHS AND LIMITATIONS OF THIS STUDY**

- A strength of this study is that the search will be designed and implemented by an experienced information specialist, followed by screening, study selection and data extraction being conducted by two investigators independently.
- A further strength is that this systematic assessment of measures of diabetic retinopathy treatment coverage can facilitate subsequent systematic reviews of the coverage of diabetic retinopathy treatment, with meta-analysis of country estimates if comparable definitions are identified.
- The studies included in the review will not be critically appraised as this review describes the methodological aspects of the measurement of diabetic retinopathy treatment coverage only.

## Introduction

 Diabetes is a large and growing issue globally.^1^ Diabetes can result in a range of complications, and diabetic retinopathy is one of the most common microvascular complications.^2^ In many countries, diabetic retinopathy is one of the leading causes of vision loss, particularly among working-age adults (20–65 years), contributing to substantial individual, family and societal impacts.^3 4^ In 2019, there was an estimated 160 million people with some degree of diabetic retinopathy, of whom 47 million had vision-threatening diabetic retinopathy.^5^

The management of diabetes, through control of blood glucose, blood pressure and lipids, is an integral part of avoiding diabetes complications, including diabetic retinopathy.^6^ Alongside the systemic control of diabetes, a further essential component of preventing vision loss from diabetic retinopathy is timely detection and direct ophthalmic treatment.^5 7^ There are four clinical stages of diabetic retinopathy: mild, non-proliferative diabetic retinopathy to moderate and severe non-proliferative diabetic retinopathy which can lead to proliferative diabetic retinopathy, the advanced stage of the disease where there is abnormal growth of new retinal blood vessels in the peripheral retina.^6^ At any stage of retinopathy, a patient may also develop diabetic macular oedema, which occurs when blood vessels leak fluid and cause swelling of the macula (which is responsible for central vision).^6^ Patients found to have moderate to proliferative diabetic retinopathy and/or diabetic macular oedema during retina screening need to be reviewed by an ophthalmologist to determine the appropriate treatment approach.^8^

The standard treatment for patients with proliferative diabetic retinopathy and/or diabetic macular oedema is one or both of laser photocoagulation and intravitreal injections of antivascular endothelial growth factor (anti-VEGF) agents, which each often require more than one treatment session.^6 8^ For patients who do not respond to these treatments, intravitreal injections of corticosteroids are often used as second-line therapy.^6 8^ In patients with advanced diabetic retinopathy, complications such as persistent vitreous haemorrhage and tractional retinal detachment can develop, and a vitrectomy may be required, where the vitreous body is removed and then replaced with another substance.^6^ Both the timely initiation and completion of the treatment plan are essential to ensure the effectiveness of diabetic retinopathy treatment in terms of halting further bleeding, slowing the growth of new blood vessels and preserving vision.^6 9^

There is evidence that, after detection of retinopathy, appropriate and timely treatment services are not accessible to all population groups.^10^,^12^ Many factors affect the extent to which people can commence and complete diabetic retinopathy treatment, and inequity in access to both systemic diabetes and retinal care is ubiquitous for some population groups, including non-dominant ethnic minorities, lower-income groups and people with low social support.^10^,^12^ Patients who are not supported to effectively manage their diabetes often have the most rapid progression of diabetic retinopathy and are less likely to receive timely retinal screening and treatment services.^8 13^ Further, in many countries, retinal screening services are integrated into primary care, available at community locations and implemented by technicians using cameras, so tend to be more accessible than treatment services which require ophthalmologists and specific equipment. Therefore, timely detection does not universally lead to timely treatment.^14^ Commonly reported barriers to treatment for diabetic retinopathy include the high cost of treatment, services being located in distant or inconvenient locations, long waiting times, being frightening and the limited capacity of the eye care workforce.^9 15^

Universal health coverage is currently a strategic priority of the World Health Organization (WHO) which aims to ensure that people receive the health services they need, of sufficient quality to be effective, without incurring financial hardship.^16^ Service coverage indicators, including treatment coverage indicators, are crucial to monitor progress towards universal health coverage.^17^ Coverage of health services captures the proportion of a population needing a service who use it.^18^ Thus, diabetic retinopathy treatment coverage indicators aim to measure the number of people requiring treatment for diabetic retinopathy and who receive it. Monitoring the coverage of diabetic retinopathy treatment services is crucial to have a thorough understanding of the extent to which services are accessible for patients to commence and complete treatment. For diabetes eye care, a retinal screening coverage indicator was included in WHO’s inaugural Eye Care Indicator Menu, while retinal treatment coverage was not.^19^ There is great variability in how studies define and report treatment coverage from routinely collected clinical records. For example, some studies report patient adherence to a first ophthalmology appointment or to commencing treatment,^20^ while others report compliance to further treatment sessions and follow-up appointments by measuring treatment gaps, delays or loss to follow-up which can affect the success of diabetic retinopathy treatment.^10 15 21^ The term treatment coverage will be used in this review to encompass all measures that report some aspect of service uptake for diabetic retinopathy treatment, including measures that quantify the patients who attended treatment sessions or follow-up appointments as well as measures that quantify non-attendance.

Treatment coverage has also been measured in population-based surveys by using retinal images of survey participants to quantify how many had evidence of diabetic retinopathy treatment.^22^ The variation in the definition of these treatment coverage measures also contribute to substantial differences in estimates reported across studies.^23 24^ In the *World Report on Vision*, WHO included the development of definitions for service coverage indicators as a key recommendation, which could include an indicator for diabetic retinopathy treatment coverage.^25^ If a definition of treatment coverage could be standardised, the monitoring of quality of diabetes eye care at the facility-level, subnational-level and national-level could more readily be compared.^23 25^

### Objective

The aim of this methodological review is to provide a comprehensive summary of how diabetic retinopathy treatment coverage has been defined in published studies from any world region and the extent to which these have been disaggregated by population groups. Diabetic retinopathy treatment coverage could be reported for one or more of: (1) laser photocoagulation; (2) intravitreal injections of anti-VEGF agents; (3) intravitreal injections of corticosteroids; (4) vitrectomy. The PROGRESS framework (place of residence, race/ethnicity/culture/language, occupation, gender/sex, religion, education, socioeconomic status and social capital) will be used to systematically assess disaggregation by population groups.^26^ This study will focus on identifying and summarising the sources of data used and the population included in the denominator and numerator population. The results of this review will be combined with that of an ongoing systematic review of retina screening coverage for people with diabetes^27^ to inform the development of an indicator to monitor effective coverage of diabetes eye care services.

## Methods

This protocol has been prepared in accordance with the guidance on the conduct and reporting of methodological studies.^28 29^ It was registered on Open Science Framework on 6 August 2024 and can be viewed online (https://osf.io/5b93m).

### Eligibility criteria

#### Population and context

We will include studies from any country reporting diabetic retinopathy treatment coverage for adults with diabetic retinopathy and/or diabetic macular oedema attributable to any type of diabetes for one or more of: (1) laser photocoagulation; (2) intravitreal injections of anti-VEGF agents; (3) intravitreal injections of corticosteroids; (4) vitrectomy. We will exclude studies reporting service coverage of ophthalmology appointments without explicit mention of one of the four treatment modalities, as well as studies that analyse clinical outcomes to determine an appropriate treatment follow-up schedule. There will be no restrictions on patient characteristics such as sex, ethnicity, duration of diabetes or location. We will include studies measuring treatment coverage for diabetic retinopathy alongside other conditions (eg, treatment coverage for anti-VEGF among people with diabetic retinopathy and/or macular degeneration) if treatment coverage for diabetic retinopathy is reported separately. We will exclude studies reporting outcomes only for people with gestational diabetes as diabetic retinopathy rarely occurs during pregnancy in this population.^30^ We will also exclude studies reporting outcomes only in children (aged 18 years or below) as there is limited evidence for treatment in paediatric patients with diabetic retinopathy and/or diabetic macular oedema and treatment is usually not required until adulthood.^31^

#### Type of studies

We will include the published (peer-reviewed) literature that report primary data. We will include prospective and retrospective cohort studies and cross-sectional studies. All editorials, protocols, reports of pilot studies and conference abstracts will be excluded. We will also exclude systematic reviews or other evidence syntheses but will examine the reference lists of relevant reviews to identify any potentially relevant studies. We will only include studies that refer to some measure of diabetic retinopathy treatment coverage in the abstract. As the use of anti-VEGF agents in the treatment of proliferative diabetic retinopathy and/or diabetic macular oedema has become more widespread in the last decade, we will limit our review to studies published since 1 January 2015.^32 33^ The search will have no language restrictions and every effort will be made to translate studies reported in languages other than English.

### Information sources and search strategy

We will include all studies published from 1 January 2015 to the search date by searching the Medline (Ovid) and Embase electronic databases using a search strategy developed and run by an experienced information specialist in July 2024. Key search terms include diabetic retinopathy, diabetic macular oedema, photocoagulation, VEGF, dexamethasone and vitrectomy. Our full search strategy for Medline and Embase is provided in online supplemental appendix 1. We will download and deduplicate the results in EndNote, and then export the results into Covidence, an online systematic review platform, (Veritas Health Innovation, Melbourne, Australia; available at www.covidence.org) for screening. We will examine the reference list of relevant reviews that we identify during the initial screening process and consider any additional potentially relevant primary studies against our inclusion criteria. As this is a methodological study of published literature, grey literature will not be searched.

### Study selection

Study selection will be performed in Covidence. Two investigators will independently screen the title and abstracts of all studies identified in the search and the full texts of all potentially relevant publications will then be acquired and assessed to establish eligibility for inclusion. Publications that do not meet the inclusion criteria will be excluded and a reason for exclusion will be assigned to each study. Any disagreements in the screening of search results will be resolved by discussion and consultation with a third investigator as needed.

### Data extraction

Data extraction will be performed in Covidence. Prior to data collection, the data extraction form will be piloted by two investigators on five studies based on the data items specified below, and modifications undertaken as required. Data will then be extracted independently by two investigators and any discrepancies will be resolved by discussion with a third investigator if necessary.

We will extract information on the study and participant characteristics, as well as detailed information on the primary outcome measure of diabetic retinopathy treatment coverage.

1. Study characteristics: title, year of publication, language of publication, country/countries of study, data source (eg, electronic vs paper-based medical records, facility-based survey data, population-based survey data, service activity or payment data), year(s) of data collection, number of participants and extent of missing data.
2. Participant and context characteristics: participant inclusion criteria (age, diabetes type), diabetic retinopathy status,^34^ health sector (eg, public/private/third sector) and financing of treatment (eg, state funded or out of pocket costs).
3. Outcome(s): we will extract details for all treatment coverage outcomes reported. For each outcome we will extract:
  - Name and definition of treatment coverage (including details on the numerator, denominator, the condition being treated and any exclusion criteria for the eligible population).
  - Treatment method (laser/anti-VEGF/corticosteroids/vitrectomy/combination of treatment methods).
  - Number of treatment sessions assessed (eg, first, all^15 20^).
  - Follow-up interval of treatment (eg, returned for follow-up appointment within 6 months^20^).
  - Measurement type (eg, cross-sectional during a defined time period,^10 15 21^ time-to-event such as, time to discontinuation^35 36^).
  - Follow-up period of study population (eg, 12 months, 5 years).
  - Disaggregation by any factors in the PROGRESS framework.^26^
  - Report of post-treatment visual acuity (yes/no).
  - Any reflections of authors on strengths or weaknesses of the treatment coverage measure.

### Synthesis of results

Data synthesis will focus on outlining the full range of definitions of diabetic retinopathy treatment coverage. We will descriptively summarise the characteristics of the studies reporting the measures, including source of data, inclusion criteria, treatment method and extent of data missingness. Categorical data (eg, number of studies using routinely collected facility-based data vs population-based survey data) will be reported as frequencies and percentages, while continuous data (eg, number of participants) will be provided as the median and IQR.

In keeping with available guidance, we will not assess risk of bias of the included studies because this review describes the methodological aspects of the measurement of diabetic retinopathy treatment coverage rather than establishing the trustworthiness of effect measures, where risk of bias assessment is arguably most useful.^28^ We do not plan to report the actual levels of diabetic retinopathy treatment coverage reported in these studies as this is beyond the scope of a methodological review; the results of this methodological review can inform subsequent systematic review and meta-analysis of country estimates of diabetic retinopathy treatment coverage.

### Patient and public involvement

Patients and the public will not be involved in the conduct of this methodological review.

### Ethics and dissemination

This review will only include published data; thus, no ethical approval will be sought. The findings of this review will be published in a peer-reviewed journal and presented at relevant conferences. The findings will also be considered in conjunction with an ongoing review on retinal screening for diabetic retinopathy^27^ to develop indicators for monitoring of services along the diabetes eye care pathway, which could include a measure of effective service coverage. It is beyond the scope of this methodological study to summarise the service coverage or outcomes of the included studies, but it may identify whether sufficient evidence is available to warrant a subsequent systematic review of one or more treatment modality.

## supplementary material

10.1136/bmjopen-2024-092081 online supplemental file 1

## References

[1] International Diabetes Federation (2021). IDF Diabetes Atlas, 10th edn.

[2] World Health Organization (2016). Global report on diabetes.

[3] Teo ZL, Tham Y-C, Yu M (2021). Global Prevalence of Diabetic Retinopathy and Projection of Burden through 2045: Systematic Review and Meta-analysis. Ophthalmology.

[4] Arun CS, Al-Bermani A, Stannard K (2009). Long-term impact of retinal screening on significant diabetes-related visual impairment in the working age population. Diabet Med.

[5] Burton MJ, Ramke J, Marques AP (2021). The Lancet Global Health Commission on Global Eye Health: vision beyond 2020. Lancet Glob Health.

[6] Wong TY, Cheung CMG, Larsen M (2016). Diabetic retinopathy. Nat Rev Dis Primers.

[7] Vujosevic S, Aldington SJ, Silva P (2020). Screening for diabetic retinopathy: new perspectives and challenges. *The Lancet Diabetes & Endocrinology*.

[8] Wong TY, Sun J, Kawasaki R (2018). Guidelines on Diabetic Eye Care: The International Council of Ophthalmology Recommendations for Screening, Follow-up, Referral, and Treatment Based on Resource Settings. Ophthalmology.

[9] Bascaran C, Mwangi N, D’Esposito F (2021). Effectiveness of interventions to increase uptake and completion of treatment for diabetic retinopathy in low- and middle-income countries: a rapid review protocol. Syst Rev.

[10] Obeid A, Gao X, Ali FS (2018). Loss to Follow-Up in Patients with Proliferative Diabetic Retinopathy after Panretinal Photocoagulation or Intravitreal Anti-VEGF Injections. Ophthalmology.

[11] Green M, Tien T, Ness S (2020). Predictors of Lost to Follow-Up in Patients Being Treated for Proliferative Diabetic Retinopathy. Am J Ophthalmol.

[12] Abdelmotaal H, Ibrahim W, Sharaf M (2020). Causes and Clinical Impact of Loss to Follow-Up in Patients with Proliferative Diabetic Retinopathy. J Ophthalmol.

[13] Lanzetta P, Sarao V, Scanlon PH (2020). Fundamental principles of an effective diabetic retinopathy screening program. Acta Diabetol.

[14] Lin S, Ramulu P, Lamoureux EL (2016). Addressing risk factors, screening, and preventative treatment for diabetic retinopathy in developing countries: a review. *Clinical Exper Ophthalmology*.

[15] Weiss M, Sim DA, Herold T (2018). Compliance and Adherence of Patients with Diabetic Macular Edema to Intravitreal Anti-Vascular Endothelial Growth Factor Therapy in Daily Practice. Retina (Philadelphia, Pa).

[16] World Health Organization (2021). International bank for reconstruction and development/the world bank. tracking universal health coverage: 2021 global monitoring report.

[17] Boerma T, AbouZahr C, Evans D (2014). Monitoring intervention coverage in the context of universal health coverage. PLoS Med.

[18] Tanahashi T (1978). Health service coverage and its evaluation. Bull World Health Organ.

[19] World Health Organization (2022). Eye Care Indicator Menu (ECIM): A Tool for Monitoring Strategies and Actions for Eye Care Provision.

[20] Bresnick G, Cuadros JA, Khan M (2020). Adherence to ophthalmology referral, treatment and follow-up after diabetic retinopathy screening in the primary care setting. BMJ Open Diabetes Res Care.

[21] Angermann R, Rauchegger T, Nowosielski Y (2019). Treatment compliance and adherence among patients with diabetic retinopathy and age-related macular degeneration treated by anti-vascular endothelial growth factor under universal health coverage. *Graefes Arch Clin Exp Ophthalmol*.

[22] Keel S, Xie J, Foreman J (2017). The Prevalence of Diabetic Retinopathy in Australian Adults with Self-Reported Diabetes: The National Eye Health Survey. Ophthalmology.

[23] Rose MA, Vukicevic M, Koklanis K (2020). Adherence of patients with diabetic macular oedema to intravitreal injections: A systematic review. *Clinical Exper Ophthalmology*.

[24] Shahzad H, Mahmood S, McGee S (2023). Non-adherence and non-persistence to intravitreal anti-vascular endothelial growth factor (anti-VEGF) therapy: a systematic review and meta-analysis. Syst Rev.

[25] World Health Organization (2019). World report on vision.

[26] O’Neill J, Tabish H, Welch V (2014). Applying an equity lens to interventions: using PROGRESS ensures consideration of socially stratifying factors to illuminate inequities in health. J Clin Epidemiol.

[27] Chabba N, Silwal PR, Bascaran C (2024). What is the coverage of retina screening services for people with diabetes? Protocol for a systematic review and meta-analysis. BMJ Open.

[28] Mbuagbaw L, Lawson DO, Puljak L (2020). A tutorial on methodological studies: the what, when, how and why. BMC Med Res Methodol.

[29] Lawson DO, Puljak L, Pieper D (2020). Reporting of methodological studies in health research: a protocol for the development of the MethodologIcal STudy reportIng Checklist (MISTIC). BMJ Open.

[30] Morrison JL, Hodgson LA, Lim LL (2016). Diabetic retinopathy in pregnancy: a review. *Clinical Exper Ophthalmology*.

[31] Sultan MB, Starita C, Huang K (2012). Epidemiology, risk factors and management of paediatric diabetic retinopathy. Br J Ophthalmol.

[32] Tan GS, Cheung N, Simó R (2017). Diabetic macular oedema. *The Lancet Diabetes & Endocrinology*.

[33] Cheung N, Wong IY, Wong TY (2014). Ocular anti-VEGF therapy for diabetic retinopathy: overview of clinical efficacy and evolving applications. Diabetes Care.

[34] World Health Organization (2018). International Classification of Diseases.

[35] Peto T, Akerele T, Sagkriotis A (2022). Treatment patterns and persistence rates with anti-vascular endothelial growth factor treatment for diabetic macular oedema in the UK: A real-world study. Diabet Med.

[36] Bakri SJ, Delyfer MN, Grauslund J (2023). Real-World Persistence and Treatment Interval in Patients with Diabetic Macular Edema Treated with Anti-Vascular Endothelial Growth Factors in the USA. Ophthalmol Ther.

