# Supplementary material for: Measures of diabetic retinopathy treatment coverage: protocol for a methodological review
Source: BMJ Open. 2025 Mar 29;15(3):e092081. doi: 10.1136/bmjopen-2024-092081 (PMC11956272; doi:10.1136/bmjopen-2024-092081)
Supplement: online supplemental file 1 [file bmjopen-15-3-s001.pdf]

Appendix 1: Search Strategy

MEDLINE

1. Diabetic Retinopathy/
2. ((diabet$ or proliferative or non-proliferative) adj4 retinopath$).tw.
3. diabetic retinopathy.kw.
4. (diabet$ adj3 (eye$ or vision or visual$ or sight$)).tw.
5. (retinopath$ adj3 (eye$ or vision or visual$ or sight$)).tw.
6. (DR adj3 (eye$ or vision or visual$ or sight$)).tw.
7. (diabet$ adj3 macula$ adj3 oedema).tw.
8. (diabet$ adj3 macula$ adj3 edema).tw.
9. (DMO or DME).tw.
10. or/1-9
11. exp light coagulation/
12. photocoagulat$.tw.
13. (photo adj1 coagulat$).tw.
14. ((focal or grid) adj3 laser$).tw.
15. (coagulat$ or argon or krypton or YAG or diode or micropulse or panretinal).tw.
16. or/11-15
17. Vascular Endothelial Growth Factor A/
18. Endothelial Growth Factors/
19. exp Angiogenesis Inhibitors/
20. (ranibizumab$ or bevacizumab$ or avastin$ or aflibercept$ or conbercept$ or OPT 302 or Opthea$ or RTH258 or faricimab or brolucizumab or leizumabor or abicipar pegol).tw.
21. (anti adj2 VEGF$).tw.
22. (anti adj1 angiogen$).tw.
23. (endothelial adj2 growth adj2 factor$).tw.
24. or/17-23
25. Dexamethasone/
26. Fluocinolone Acetonide/
27. Triamcinolone Acetonide/
28. (dexamethasone or fluocinolone or triamcinolone).tw.
29. or/25-28
30. Vitrectomy/
31. vitrectom$.tw.
32. PPV$.tw.
33. or/30-32
34. 16 or 24 or 29 or 33
35. 10 and 34
36. cohort studies/
37. cross-sectional studies/
38. prospective studies/ or retrospective studies/
39. (prospective$ or retrospective$ or cohort or crossectional).tw.
40. (cross adj1 section$).tw.
41. or/36-40

42. 35 and 41
43. limit 42 to yr="2015 -Current"
44. limit 43 to (comment or editorial or letter or personal narrative)
45. 43 not 44

Embase

1. diabetic retinopathy/
2. ((diabet$ or proliferative or non-proliferative) adj4 retinopath$).tw.
3. diabetic retinopathy.kw.
4. (diabet$ adj3 (eye$ or vision or visual$ or sight$)).tw.
5. (retinopath$ adj3 (eye$ or vision or visual$ or sight$)).tw.
6. (DR adj3 (eye$ or vision or visual$ or sight$)).tw.
7. diabetic macular edema/
8. (diabet$ adj3 macula$ adj3 oedema).tw.
9. (diabet$ adj3 macula$ adj3 edema).tw.
10. (DMO or DME).tw.
11. or/1-10
12. laser coagulation/
13. photocoagulat$.tw.
14. (photo adj1 coagulat$).tw.
15. ((focal or grid) adj3 laser$).tw.
16. (coagulat$ or argon or krypton or YAG or diode or micropulse or panretinal).tw.
17. or/12-16
18. endothelial cell growth factor/
19. angiogenesis inhibitor/
20. (ranibizumab$ or bevacizumab$ or avastin$ or aflibercept$ or conbercept$ or OPT 302 or Opthea$ or RTH258 or faricimab or brolucizumab or leizumabor or abicipar pegol).tw.
21. (anti adj2 VEGF$).tw.
22. (anti adj1 angiogen$).tw.
23. (endothelial adj2 growth adj2 factor$).tw.
24. or/18-23
25. dexamethasone/
26. fluocinolone acetonide/
27. triamcinolone acetonide/
28. (dexamethasone or fluocinolone or triamcinolone).tw.
29. or/25-28
30. exp vitrectomy/
31. vitrectom$.tw.
32. PPV$.tw.
33. or/30-32
34. 17 or 24 or 29 or 33
35. 11 and 34
36. cohort analysis/
37. cross-sectional study/
38. prospective study/
39. retrospective study/
40. (prospective$ or retrospective$ or cohort or crossectional).tw.

41. (cross adj1 section$).tw.
42. or/36-41
43. 35 and 42
44. limit 43 to conference abstract status
45. 43 not 44
46. limit 45 to yr="2015 -Current"
47. limit 46 to (conference paper or "conference review" or editorial or letter or note)
48. 46 not 47
