# Supplementary material for: Measures of diabetic retinopathy treatment coverage: protocol for a methodological review
Source: BMJ Open. 2025 Mar 29;15(3):e092081. doi: 10.1136/bmjopen-2024-092081 (PMC11956272; doi:10.1136/bmjopen-2024-092081)
Supplement: Supplementary file 2 [file bmjopen-2024-092081.reviewer_comments.pdf]

PEER REVIEW HISTORY

BMJ Open publishes all reviews undertaken for accepted manuscripts. Reviewers are asked to complete a checklist review form and are provided with free text boxes to elaborate on their assessment. These free text comments are reproduced below.

ARTICLE DETAILS

Title (Provisional)

Measures of diabetic retinopathy treatment coverage: Protocol for a methodological review

Authors

Chabba, Nimisha; Silwal, Pushkar Raj; Bascaran, Covadonga; Murphy, Rinki; Gordon, Iris; Mwangi, Nyawira; Bhatta, Subash; Pant, Nayana; Burton, Matthew J; Keel, Stuart; Evans, Jennifer; Ramke, Jacqueline

---

VERSION 1 - REVIEW

**Reviewer**	**1**
**Name**	**James, Marilyn**
**Affiliation**	**University of Nottingham, School of Medicine**
**Date**	**09-Sep-2024**
**COI**	**No competing interests**

Measures of diabetic retinopathy treatment coverage: Protocol for a methodological review

The authors raise an important question re lack of follow up and agreement on successful treatment coverage and as such want to review and consider a “standardised” way this should be done. I think the “methodological review” in the title is misleading and should be removed and re written as its not about studying methods its about studying what to measure and agreeing a common outcome set.

P3 treatment choice 1-4 make good sense but treatment option 5 (and only if linked to 1-4) doesn’t and is confusing and detracts from the central message, I think it would be clearer if this was dropped and 1-4 only used.

The work could benefit from including an earlier start date than 2015

A limitation as indicated by the authors is lack of validation of quality of the outcome measurement. This limitation could be expressed more clearly than “The studies included in the review will not be critically appraised” P4

P5 the authors make an excellent point that outcome of treatment can be adversely affected by access to other and good quality diabetic care.

The authors outline important factors of coverage and adherence / compliance and that both affect end point outcomes. This is an important point and well picked up, but then not capitalised on later in the work. This explanation re almost the 2 stages of outcome could have beneficially more explanation if space allowed.

P6 ln 30-31 Objective: "how diabetic retinopathy treatment coverage has been defined in published". I feel the term "coverage" should be dropped from the objective. The work is basically about how its measured and what is measured. The use of coverage here is confusing in the context of the previous section which discusses access to treatment and compliance.

The exclusions are sensible.

I'm not familiar with "Covidence" others may also not be and perhaps needs an explanatory sentence.

P8 outcome "h" I feel this does not go far enough. It should record more than just visual acuity reported or not, but where this is measured, include the VA or range or important difference.

P8 ln 42-43 given the authors talk about coverage at the start of the protocol, and they will know this, I would think it is indeed worth reporting the actual levels of treatment coverage rather than saying "We do not plan to report the actual levels of diabetic retinopathy treatment coverage"

Reviewer 2

Name Sasongko, M. B.

Affiliation Universitas Gadjah Mada Fakultas Kedokteran Kesehatan Masyarakat dan Keperawatan, Ophthalmology

Date 11-Sep-2024

COI None

This is interesting and straight forward protocol paper by Chabba and colleagues. I can see the value of this protocol paper, and the importance of the ongoing review paper, if happening next.

The paper is well-written, clear, and concise. I would suggest to include detailed the search strategy (as in the supplementary appendix as part of this main paper.

Reviewer 3

Name Mohan, Viswanathan

Affiliation Madras Diabetes Research Foundation, Diabetology

Date 01-Feb-2025

COI **none**

The manuscript is the protocol of methodology for a systematic review of diabetic retinopathy (DR) treatment from globally published studies. The authors have written the methodology for the systematic review with the inclusion and exclusion criteria and how the published texts / data extraction will be performed in Covidence.

1. Although you have aptly mentioned that you would be excluding children (below 18 years) as DR requiring Rx would be low in the group, it would be important to include published studies of treatment of DR in both type 1 diabetes and type 2 diabetes, although DR treatment studies in type 1 diabetes may be a smaller number.
2. In studies related to intravitreal anti-VEGF agents, kindly also include studies on biosimilars anti-VEGF agents used for treatment of diabetic macular oedema.
3. Kindly ensure that studies from across the world are considered to reflect on the treatment practices across various countries with different economies, as there is a significant cost to DR treatment- some out of the pocket and some covered by government / insurance.
4. Studies on the role of systemic risk factors (glycemic control, blood pressure, lipid profile etc) in diabetic retinopathy (DR) treatment can be included too.

VERSION 1 - AUTHOR RESPONSE

**Comments to the author**	**Author response**	**Changes in the manuscript**
Reviewer 1: Prof. Marilyn James, University of Nottingham		
The authors raise an important question re lack of follow up and agreement on successful treatment coverage and as such want to review and consider a "standardised" way this should be done. I think the "methodological review" in the title is misleading and should be removed and re written as its not about studying	Thank you for this feedback, which reflects an observation made in the best resources available on methodological studies that we draw on and reference in the protocol[1,2] regarding a lack of consensus on what these types of evidence syntheses are called. These guiding papers outline a range of terms historically used and suggest "methodological" is best suited and that these studies "evaluate the design, conduct, analysis and reporting of other studies and where the unit of analysis is the research report".<br><br>Because our objective (to summarise the definition of outcomes reported in other studies), aligns with the definition of a	No changes made.

methods its about studying what to measure and agreeing a common outcome set.	methodological study, we would prefer to remain in keeping with the guiding documents.<br><br>Thus, we have not made any change in response to this comment.<br><br>[1]Mbuagbaw L, Lawson DO, Puljak L, Allison DB, Thabane L. A tutorial on methodological studies: the what, when, how and why. BMC Medical Research Methodology 2020;20(1):1-12.<br>[2]Lawson DO, Puljak L, Pieper D, Schandelmaier S, Collins GS, Brignardello-Petersen R, . . . Samaan Z. Reporting of methodological studies in health research: a protocol for the development of the MethodologIcal STudy reportIng Checklist (MISTIC). BMJ open 2020;10(12):e040478.	
P3 treatment choice 1-4 make good sense but treatment option 5 (and only if linked to 1-4) doesn't and is confusing and detracts from the central message, I think it would be clearer if this was dropped and 1-4 only used.	Thank you for your helpful feedback. We have removed treatment option #5 and have updated the text on page 2, lines 19-20; page 4, lines 31-32 and page 5, lines 6-7.	Page 2, lines 19-20:<br>We will include studies from any world region reporting diabetic retinopathy treatment coverage for one or more of: (1) laser photocoagulation; (2) intravitreal injections of anti-vascular endothelial growth factor agents; (3) intravitreal injections of corticosteroids; **and** (4) vitrectomy.~~,~~ ~~**and (5) ophthalmological review of people with diagnosed diabetic retinopathy (if reported in conjunction with any of treatment (1) to (4)).**~~<br><br>Page 4, lines 31-32:<br>Diabetic retinopathy treatment coverage could be reported for one or more of: (1) laser photocoagulation; (2) intravitreal injections of anti-VEGF agents; (3) intravitreal injections of corticosteroids; **and** (4) vitrectomy.~~,~~ ~~**and (5) ophthalmological review (if reported in conjunction with any of treatment (1) to (4)).**~~<br><br>Page 5, lines 6-7:<br>We will include studies from any country reporting diabetic retinopathy treatment coverage for adults with diabetic retinopathy and/or diabetic macular oedema attributable to any type of diabetes for one or more of: (1) laser photocoagulation; (2) intravitreal injections of anti-VEGF agents; (3) intravitreal injections of corticosteroids; **and** (4) vitrectomy~~,~~ ~~**and (5) ophthalmological review**~~

		~~(if reported in conjunction with any of treatment (1) to (4)).~~
The work could benefit from including an earlier start date than 2015	Thank you for this suggestion. While we recognise we could provide an exhaustive summary of the literature on this topic, as outlined in the protocol, we chose 2015 to capture the uptake of anti-VEGF over the last decade, given it is now a mainstay of treatment. In addition, as outlined in our submission, the broader project that this synthesis will inform is forward looking to establish indicators for effective DR services, so we wish to include definitions from the most contemporary literature.	No change made.
A limitation as indicated by the authors is lack of validation of quality of the outcome measurement. This limitation could be expressed more clearly than "The studies included in the review will not be critically appraised" P4	We thank the reviewer for this helpful comment. We have now added the following text to explain our reasoning for this to page 6, lines 41-44. As outlined in the available guidance by Mbuagbaw and colleagues, the intrinsic value of risk of bias in methodological studies is not obvious, which we have now made clearer.	Page 6, lines 41-44: **In keeping with available guidance, w**e will not assess risk of bias of the included studies because this review describes the methodological aspects of the measurement of diabetic retinopathy treatment coverage ~~only~~ **rather than establishing the trustworthiness of effect measures, where risk of bias assessment is arguably most useful [28].**
P5 the authors make an excellent point that outcome of treatment can be adversely affected by access to other and good quality diabetic care.	Thank you so much.	-
The authors outline important factors of coverage and adherence / compliance and that both affect end point outcomes. This is an important point and well picked up, but then not capitalised on later in the work. This explanation re almost the 2 stages of outcome could have beneficially more explanation if space allowed.	Many thanks for this positive feedback. This is certainly a key topic we aim to elaborate on in the review itself. We are pleased we have effectively outlined it here and will certainly expand on it in the context of the papers that meet our inclusion criteria.	-
P6 ln 30-31 Objective: "how diabetic retinopathy treatment coverage has been	Thank you for your feedback. The broader project within which this review sits aligns with priorities of the World Health Organization, including Universal	Page 3, lines 44-45 and page 4, lines 1-5: **Universal Health Coverage is currently a strategic priority of the**

defined in published". I feel the term "coverage" should be dropped from the objective. The work is basically about how its measured and what is measured. The use of coverage here is confusing in the context of the previous section which discusses access to treatment and compliance.	Health Coverage and the need to improve effective coverage of eye health services as outlined in the inaugural World Report on Vision in 2019. We appreciate the terminology may not be familiar to readers of this protocol, so have expanded our text briefly to make this clearer. We have added the following text to page 3, lines 44-45 and page 4, lines 1-5.	**World Health Organization (WHO) and aims to ensure that all people receive the health services they need, of sufficient quality to be effective, without incurring financial hardship [16]. Service coverage indicators, including treatment coverage indicators, are crucial to monitor progress towards Universal Health Coverage [17]. Coverage of health services captures the proportion of a population needing a service who use it [18]. Thus, diabetic retinopathy treatment coverage indicators aim to measure the number of people requiring treatment for diabetic retinopathy who receive it.** Monitoring the coverage of diabetic retinopathy treatment services is crucial to have a thorough understanding of the extent to which services are accessible for patients to commence and complete treatment.
The exclusions are sensible.	Thank you.	-
I'm not familiar with "Covidence" others may also not be and perhaps needs an explanatory sentence.	Thank you for this suggestion. We have added the following text to page 5, line 35-36.	Page 5, line 35-36:<br>We will download and de-duplicate the results in EndNote, and then export the results into Covidence, **an online systematic review platform**, (Veritas Health Innovation, Melbourne, Australia; available at www.covidence.org) for screening.
P8 outcome "h" I feel this does not go far enough. It should record more than just visual acuity reported or not, but where this is measured, include the VA or range or important difference.	Many thanks for this suggestion. While we agree this is important information, it would take our review into a different evidence synthesis type, with implications for several components of our methodological approach. Our methods currently align with available guidance on methodological studies which we would like to retain. To recognise the importance of this information, we have added text to the Dissemination section on page 7, lines 10-13	Page 7, lines 10-13:<br>It is beyond the scope of this methodological study to summarise the service coverage or outcomes of the included studies, but it may identify whether sufficient evidence is available to warrant a subsequent systematic review of one or more treatment modality.
P8 ln 42-43 given the authors talk about coverage at the start of the protocol, and they will know this, I would think it is indeed worth	Thank you for your suggestion, which aligns with the suggestion directly above. For the same reason provided above, we believe we are unable to modify the approach, but have included treatment coverage in our added text in the	Page 6, lines 45 and page 7, line 1:<br>We do not plan to report the actual levels of diabetic retinopathy treatment coverage reported in these studies **as this is beyond the scope of a methodological**

reporting the actual levels of treatment coverage rather than saying "We do not plan to report the actual levels of diabetic retinopathy treatment coverage"	Dissemination section on page 7, lines 10-13. In addition, we have added a more explicit statement to the text you refer to page 6, line 45 and page 7, line 1.	**review**; the results of this methodological review can inform subsequent systematic review and meta-analysis of country estimates of diabetic retinopathy treatment coverage.
Reviewer 2: Dr. M. B. Sasongko, Universitas Gadjah Mada Fakultas Kedokteran Kesehatan Masyarakat dan Keperawata		
This is interesting and straight forward protocol paper by Chabba and colleagues. I can see the value of this protocol paper, and the importance of the ongoing review paper, if happening next.<br>The paper is well-written, clear, and concise. I would suggest to include detailed the search strategy (as in the supplementary appendix as part of this main paper	Thank you for your helpful suggestion. We have now included key search terms in the main manuscript; however, as we understand is standard practice for protocols of evidence synthesis, the full search strategy will still be detailed in the supplementary appendix.	Page 5, lines 32-34:<br>We will include all studies published from 1 January 2015 to the search date by searching the Medline (Ovid) and Embase electronic databases using a search strategy developed and run by an experienced information specialist in July 2024. **Key search terms include diabetic retinopathy, diabetic macular oedema, photocoagulation, vascular endothelial growth factor, dexamethasone and vitrectomy.** Our full search strategy for **Medline and Embase** is provided in Supplemental Appendix 1.
Reviewer 3: Dr. Viswanathan Mohan, Madras Diabetes Research Foundation		
The manuscript is the protocol of methodology for a systematic review of diabetic retinopathy (DR) treatment from globally published studies. The authors have written the methodology for the systematic review with the inclusion and exclusion criteria and how the published texts / data extraction will be performed in Covidence.<br>1. Although you have aptly mentioned that you would be excluding children (below 18 years) as DR requiring Rx would be low in the group, it would be important to include published	Thank you for your feedback. We agree it is important to include people with type 1 diabetes, and had explicitly considered this in our criteria (page 5, lines 3-4): "We will include studies from any country reporting diabetic retinopathy treatment coverage for adults with diabetic retinopathy and/or diabetic macular oedema **attributable to any type of diabetes** for one or more of…"	No change – already within criteria.

studies of treatment of DR in both type 1 diabetes and type 2 diabetes, although DR treatment studies in type 1 diabetes may be a smaller number.		
In studies related to intravitreal anti-VEGF agents, kindly also include studies on biosimilars anti-VEGF agents used for treatment of diabetic macular oedema	Thank you for this helpful feedback. As outlined in our existing criteria (page 5, line 5) we will include studies reporting diabetic retinopathy treatment coverage for any form of intravitreal injections of anti-VEGF agents, which can include studies using biosimilar anti-VEGF agents.	No change – already within criteria.
Kindly ensure that studies from across the world are considered to reflect on the treatment practices across various countries with different economies, as there is a significant cost to DR treatment- some out of the pocket and some covered by government / insurance.	Thank you for your helpful suggestion. We have specified in our eligibility criteria that we will include studies from any country reporting diabetic retinopathy treatment coverage for adults with diabetic retinopathy and/or macular oedema on page 5, line 3. This is to ensure that we include studies from various health system settings and economies. If available in the included studies, we will also extract information on the health sector setting and financing of treatment as outlined on page 6, lines 15-16.	No change – already within criteria.
Studies on the role of systemic risk factors (glycemic control, blood pressure, lipid profile etc) in diabetic retinopathy (DR) treatment can be included too.	Thank you for this suggestion, which is certainly an important topic. Unfortunately, because it is quite removed from the work that this review will inform (i.e. the development of service coverage indicators for diabetic retinopathy treatment), we feel it is not relevant to broaden the scope to include these studies and have not made this change.	No change made.
